# Association of the rs1346044 Polymorphism of the Werner Syndrome Gene *RECQL2* with Increased Risk and Premature Onset of Breast Cancer

**DOI:** 10.3390/ijms161226192

**Published:** 2015-12-10

**Authors:** Karin Zins, Barbara Frech, Eva Taubenschuss, Christian Schneeberger, Dietmar Abraham, Martin Schreiber

**Affiliations:** 1 Laboratory for Molecular Cellular Biology, Center for Anatomy and Cell Biology, Medical University of Vienna, A-1090 Vienna, Austria; karin.zins@meduniwien.ac.at (K.Z.); dietmar.abraham@meduniwien.ac.at (D.A.); 2 Department of Obstetrics and Gynecology, Medical University of Vienna, Waehringer Guertel 18-20, A-1090 Vienna, Austria; barbara.frech@gmx.net (B.F.); eva.taubenschuss@gmx.at (E.T.); christian.schneeberger@meduniwien.ac.at (C.S.); 3 Comprehensive Cancer Center (CCC), Medical University of Vienna, A-1090 Vienna, Austria

**Keywords:** breast cancer, Werner syndrome, WRN, RECQL2, rs1346044, rs3087425, single nucleotide polymorphism (SNP)

## Abstract

Like other RECQ helicases, WRN/RECQL2 plays a crucial role in DNA replication and the maintenance of genome stability. Inactivating mutations in *RECQL2* lead to Werner syndrome, a rare autosomal disease associated with premature aging and an increased susceptibility to multiple cancer types. We analyzed the association of two coding single-nucleotide polymorphisms in *WRN*, Cys1367Arg (rs1346044), and Arg834Cys (rs3087425), with the risk, age at onset, and clinical subclasses of breast cancer in a hospital-based case-control study of an Austrian population of 272 breast cancer patients and 254 controls. Here we report that the rare homozygous CC genotype of rs1346044 was associated with an approximately two-fold elevated breast cancer risk. Moreover, patients with the CC genotype exhibited a significantly increased risk of developing breast cancer under the age of 55 in both recessive and log-additive genetic models. CC patients developed breast cancer at a mean age of 55.2 ± 13.3 years and TT patients at 60.2 ± 14.7 years. Consistently, the risk of breast cancer was increased in pre-menopausal patients in the recessive model. These findings suggest that the CC genotype of WRN rs1346044 may contribute to an increased risk and a premature onset of breast cancer.

## 1. Introduction

Breast cancer is the most common cancer of women and the exploration of its causes and the biological complexity of this disease is an issue of public health importance [1,2]. Taking into account that the integrity of the genome is compromised in almost all cancers, it has been speculated that factors contributing to genetic stability such as the *RECQL2* gene product might play a role in cancer, including breast cancer [3].

*RECQL2* belongs to the RECQ helicase gene family and plays a key role in DNA replication, recombination, telomere preservation, and repair [4,5,6,7]. The primary function of RECQL2 may be to repair DNA double-strand breaks by homologous recombination, which is essential to maintain genome integrity [8]. Therefore, RECQL2 has been proposed to function as a “caretaker” of the genome, whose dysfunction is associated both with defective DNA repair and aging- and cancer-related phenotypes [9].

Mutations in human *RECQL2* give rise to Werner syndrome (WS; OMIM: 277700), a rare autosomal recessive genetic disorder [10,11]. WS is characterized by genetic instability and the premature onset of disorders related to aging, such as graying and loss of hair, osteoporosis, atherosclerosis, diabetes mellitus, ocular cataracts, and cancer [8,11,12,13]. The spectrum of cancers is characterized by malignancies of mesenchymal origin, such as soft tissue and bone sarcomas [14]. WS is typically associated with nonsense, splicing, or frameshift mutations of *RECQL2* with missense mutations being extremely rare [15], perhaps because WS only arises when both the DNA helicase and DNA exonuclease functions of RECQL2 are lost [16]. The rare single nucleotide polymorphism (SNP) Arg834Cys within the helicase domain of *RECQL2*, was shown to be associated with a reduced WRN helicase activity, while two common SNPs of *RECQL2*, Leu1074Phe, and Cys1367Arg, exhibited essentially wild-type-level helicase and exonuclease activities [17].

In cancer research, rs1346044 leading to the non-conservative Cys1367Arg substitution has been a focus of attention [3,18,19,20]. In an earlier study, rs1346044 was identified as a risk factor for familial breast cancer in a German patient population [19]. In contrast, no association of rs1346044 with breast cancer risk was found in a Taiwanese population, which may reflect disparities between different genetic backgrounds and populations [3].

We were therefore interested to determine whether two coding polymorphisms in *RECQL2*, the common rs1346044 (Cys1367Arg) and the rare rs3087425 (Arg834Cys) are associated with the risk, age at onset, and clinical subclasses of breast cancer in Austrian women in a hospital-based case-control study. Our data suggest a critical impact of rs1346044 on breast cancer biology in the investigated Austrian population.

## 2. Results

### 2.1. Association between RECQL2 rs1346044 (Cys1367Arg) and Breast Cancer Risk

Two single nucleotide polymorphisms (SNPs) in *RECQL2* were investigated in a hospital-based case-control study. SNP rs3087425 (Arg834Cys) located in the helicase domain was successfully genotyped in 269 breast cancer patients and 249 female controls, which all exhibited the common homozygote genotype (CC; Arg/Arg). This SNP was thus non-polymorphic in our study population, precluding any further analyses. SNP rs1346044 (Cys1367Arg) in exon 34 was successfully genotyped in 526 individuals (272 patients and 254 controls). The minor allele frequency was 28.1% in patients and 24.6% in control subjects. Both the controls (*p* = 0.18) and the patients (*p* = 0.96) were in Hardy-Weinberg equilibrium. Table 1 shows the clinical characteristics of the study population, and the frequency of rs1346044 genotypes in the study population and subpopulations thereof. The fraction of patients with one or two C-alleles was significantly increased in the 68 patients with pT2-4tumors (>2 cm). 36 of these patients exhibited the TC genotype, and another four the CC (Arg/Arg) genotype (Table 1). Together, these are 58.8% of the pT2-4 patients, compared to 38.5% of pT1 patients (40 TC plus 12 CC out of 135 total; *p* = 0.005; see also Table 1). Likewise, the fraction of patients with one or two C-alleles was increased in patients with a younger age at onset. 55.5% of patients younger than 55 years exhibited either the TC (48/108) or the CC genotype (12/108), compared to 43.9% of patients over 55 years (63/164 TC plus 9/164 CC; *p* = 0.084; see next section results, below). The lymph node status was equally distributed for all genotypes (Table 1).

Next, odds ratios and 95% confidence intervals were determined to assess the breast cancer risk associated with the different rs1346044 genotypes, and with the C and T alleles. These comparisons showed non-significantly increased odds ratios between 1.08 and 1.91 (Table 2). Since our descriptive statistics indicated an association of the Cys1367Arg SNP with age at breast cancer onset (Table 1), we also determined age-adjusted odds ratios and 95% confidence intervals in addition to the unadjusted values. This increased the magnitude of the observed effects, and borderline significance was obtained for comparisons of the rare CC genotype with the other genotypes (Table 2).

**Table 1:** Frequency of the Werner Syndrome gene (*WRN*) Cys1367Arg genotypes in the study population.

	Total		TT		TC		CC	
All subjects	526		280	(53.2%)	214	(40.7%)	32	(6.1%)
Patients	272		140	(51.5%)	111	(40.8%)	21	(7.7%)
Controls	254		140	(55.1%)	103	(40.6%)	11	(4.3%)
**Patient Subgroups**								
Age (years)	<55	108	48	(44.4%)	48	(44.4%)	12	(11.1%)
	≥55	164	92	(56.1%)	63	(38.4%)	9	(5.5%)
Menopausal status	pre	63	31	(49.2%)	24	(38.1%)	8	(12.7%)
	post	173	93	(53.8%)	70	(40.5%)	10	(5.8%)
	na	36	16	(44.4%)	17	(47.2%)	3	(8.3%)
Tumor size	pT1	135	83	(61.5%)	40	(29.6%)	12	(8.9%)
	pT2-4	68	28	(41.2%)	36	(52.9%)	4	(5.9%)
	other, na	69	29	(42.0%)	35	(50.7%)	5	(7.2%)
Tumor type	ductal	152	80	(52.6%)	59	(38.8%)	13	(8.6%)
	lobular	48	28	(58.3%)	18	(37.5%)	2	(4.2%)
	other, na	72	32	(44.4%)	34	(47.2%)	6	(8.3%)
Stage	0, I	116	66	(56.9%)	40	(34.5%)	10	(8.6%)
	II–IV	93	43	(46.2%)	43	(46.2%)	7	(7.5%)
	other, na	63	31	(49.2%)	28	(44.4%)	4	(6.3%)
Grade	pG1-2	160	81	(50.6%)	68	(42.5%)	11	(6.9%)
	pG3	91	49	(53.8%)	34	(37.4%)	8	(8.8%)
	na	21	10	(47.6%)	9	(42.9%)	2	(9.5%)
Lymph node status	pN0	147	78	(53.1%)	57	(38.8%)	12	(8.2%)
	pN+	55	29	(52.7%)	21	(38.2%)	5	(9.1%)
	na	70	33	(47.1%)	33	(47.1%)	4	(5.7%)
ER status	pos	202	108	(53.5%)	77	(38.1%)	17	(8.4%)
	neg	57	29	(50.9%)	25	(43.9%)	3	(5.3%)
	na	13	3	(23.1%)	9	(69.2%)	1	(7.7%)
PR status	pos	142	73	(51.4%)	60	(42.3%)	9	(6.3%)
	neg	117	64	(54.7%)	42	(35.9%)	11	(9.4%)
	na	13	3	(23.1%)	9	(69.2%)	1	(7.7%)
HER2 status	pos	53	29	(54.7%)	19	(35.8%)	5	(9.4%)
	neg	203	106	(52.2%)	83	(40.9%)	14	(6.9%)
	na	16	5	(31.3%)	9	(56.3%)	2	(12.5%)
p53 status	pos	57	28	(49.1%)	27	(47.4%)	2	(3.5%)
	neg	195	106	(54.4%)	74	(37.9%)	15	(7.7%)
	na	20	6	(30.0%)	10	(50.0%)	4	(20.0%)

Numbers of patients in each of the indicated subgroups are shown. Numbers in parentheses indicate the fraction of patients in percent with the corresponding genotypes CC, TC, and TT, respectively. na, status not available; ER, estrogen receptor; PR, progesterone receptor; HER2, human EGF receptor 2; pre, pre-menopausal; post, post-menopausal; neg, negative; pos, positive.

**Table 2:** Association of *WRN* Cys1367Arg genotypes and alleles with breast cancer risk.

Genotypes/Alleles	Unadjusted			Adjusted for Age		
	OR	95% CI	*p*-Value	OR	95% CI	*p*-Value
CC *vs.* TT	1.91	0.89–4.11	0.116	2.26	0.98–5.24	0.050
CC *vs.* TC	1.77	0.82–3.98	0.157	2.05	0.88–4.77	0.091
TC *vs.* TT	1.08	0.75–1.54	0.683	1.09	0.73–1.63	0.679
CC + TC *vs.* TT	1.16	0.82–1.63	0.402	1.20	0.81–1.77	0.362
CC *vs.* TC + TT	1.85	0.87–3.91	0.101	2.18	0.96–4.95	0.058
C *vs.* T	1.21	0.91–1.60	0.185	1.27	0.93–1.75	0.135

Analyses of breast cancer cases *vs.* controls of the indicated genotypes or alleles are shown. OR, odds ratios; 95% CI, 95% confidence intervals.

For example, the unadjusted odds ratio for the analysis of CC compared to TT was 1.91 (95% CI, 0.89–4.11; *p* = 0.116), whereas the odds ratio adjusted for age was 2.26 (95% CI, 0.98–5.25; *p* = 0.05; Table 2). Although odds ratios associated with the CC genotype were between 1.77 and 2.26 indicating an approximately doubled risk of breast cancer, these observations did not quite attain significance at the *p* < 0.05 level, which may be due to the small number of CC patients (Table 2). Specifically, age-adjusted odds ratios for breast cancer risk of the CC genotype were 2.26 (95% CI, 0.98–5.24; *p* = 0.05) compared to the common homozygotes (TT), 2.05 (95% CI, 0.88–4.77; *p* = 0.09) compared to the heterozygote (TC), and 2.18 (95% CI, 0.96–4.95; *p* = 0.06) compared to TT and TC combined (Table 2).

Table 3 shows the odds ratios for rs1346044 in selected breast cancer subpopulations. After fitting dominant, recessive, and log-additive inheritance models, statistically significant effects were identified (Table 3). The odds ratio associated with an early breast cancer onset under the age of 55 years was 1.54 (95% CI, 0.98–2.41; *p* = 0.063) under a dominant genetic model, 2.76 (95% CI, 1.18–6.47; *p* = 0.021) under a recessive genetic model, and 1.58 (95% CI, 1.10–2.27; *p* = 0.014) under a log-additive genetic model. Thus, rs1346044 was significantly associated with an increased breast cancer risk in patients under 55 years based on recessive and log-additive genetic models and almost reached statistical significance based on a dominant model (Table 3; see also next section results, below). A similar result was obtained in pre-menopausal patients, which overlap to a large extent with the patients under age 55, under a recessive model with a statistically significant odds ratios (OR) of 3.21 (95% CI, 1.23–8.36; *p* = 0.022; Table 3). Furthermore, the C-allele was significantly associated with large tumors (pT2–pT4; >2cm), as indicated by an odds ratio of 1.75 (95% CI, 1.02–3.02, *p* = 0.041) based on a dominant genetic model, and almost reached statistical significance based on a log-additive model (OR 1.55; 95% CI, 0.99–2.42; *p* = 0.055; Table 3). A trend for an increased risk of estrogen receptor (ER) positive breast cancer was observed under a recessive model (OR 2.03; 95% CI, 0.93–4.44; *p* = 0.072; Table 3). In contrast, a trend for an increased risk of PR negative breast cancer was observed, as indicated by an odds ratio of 2.29 (95% CI, 0.96–5.45; *p* = 0.063) under a recessive model (Table 3). However, none of these receptor associations was statistically significant at a level of *p* < 0.05.

### 2.2. rs1346044 and Age at Breast Cancer Onset

**Figure 1:**
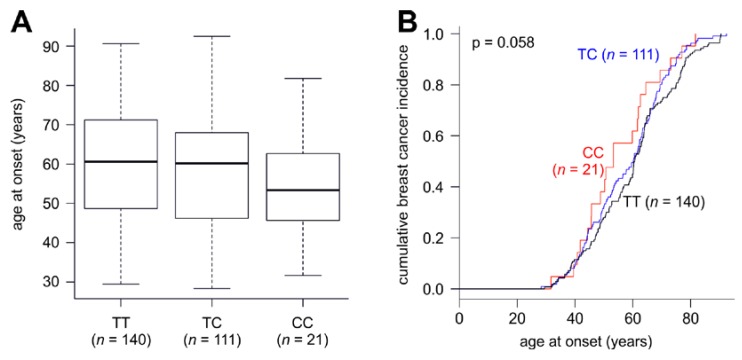
Age at breast cancer onset according to Cys1376Arg genotypes of *WRN* (Werner Syndrome gene). (**A**) Boxplot of the age at onset of patients with the genotypes TT, TC, and CC; (**B**) Curves of the cumulative breast cancer incidence at the indicated ages at onset of patients with the genotypes TT, TC and CC.

We found the minor C allele to be significantly associated with an increased breast cancer risk in younger patients (<55 years) in recessive and log-additive inheritance models (Table 3). Moreover, 12 out of 21 CC patients (57.1%), 48 out of 111 TC patients (43.2%), but only 48 out of 140 TT patients (34.3%) had developed breast cancer by the age of 55 (see also Table 1). Accordingly, we analyzed the association of these genotypes with the age at breast cancer diagnosis further. TT patients were diagnosed with breast cancer at a mean age of 60.2 ± 14.7 years (median, 60.6 years) and CC patients at a mean age of 55.2 ± 13.3 years (median, 53.3 years; Figure 1A). Thus, CC patients had a >5 years younger age at onset than TT patients. However, this effect was not significant (*p* = 0.12, unpaired two-sided *t*-test), which may, at least in part, be due to the small number of CC patients. TC patients exhibited an intermediate mean age at onset (58.1 ± 13.7 years; median, 60.2 years; Figure 1). Curves of the cumulative breast cancer incidence also showed a considerably earlier age at breast cancer onset of CC patients, which was of borderline significance (*p* = 0.058, log-rank test; Figure 1B).

**Table 3:** Association of the WRN Cys1367Arg single nucleotide polymorphism (SNP) with breast cancer risk in patient subgroups.

Cys1367Arg (rs1346044)	Patients			Dominant Model			Recessive Model			Log-Additive Model		
	Subgr.	No.	Percent	OR	95% CI	*p*	OR	95% CI	*p*	OR	95% CI	*p*
Age (years) ^a^	<55	108	39.7%	1.54	0.98–2.41	0.063	2.76	1.18–6.47	0.021	1.58	1.10–2.27	0.014
	≥55	164	60.3%	0.96	0.65–1.43	0.844	1.28	0.52–3.17	0.591	1.01	0.72–1.40	0.976
Menopausal status	pre	63	26.7%	1.27	0.73–2.20	0.400	3.21	1.23–8.36	0.022	1.45	0.93–2.25	0.100
	post	173	73.3%	1.06	0.72–1.56	0.782	1.36	0.56–3.26	0.500	1.08	0.78–1.50	0.629
Tumor type	ductal	152	76.0%	1.20	0.86–1.67	0.282	2.07	0.90–4.74	0.086	1.20	0.86–1.67	0.282
	lobular	48	24.0%	0.88	0.47–1.64	0.680	0.96	0.21–4.48	0.959	0.90	0.53–1.55	0.710
Tumor size	pT1	135	66.5%	0.77	0.50–1.18	0.226	2.16	0.92–5.02	0.077	0.95	0.67–1.34	0.780
	pT2-4	68	33.5%	1.75	1.02–3.02	0.041	1.38	0.43–4.48	0.600	1.55	0.99–2.42	0.055
Stage	0 or I	116	55.5%	0.93	0.60–1.45	0.749	2.08	0.86–5.06	0.109	1.07	0.75–1.54	0.711
	II–IV	93	44.5%	1.43	0.89–2.30	0.142	1.80	0.68–4.79	0.252	1.40	0.94–2.07	0.096
Grade	pG1-2	160	63.7%	1.20	0.81–1.78	0.372	1.63	0.69–3.85	0.267	1.22	0.88–1.69	0.244
	pG3	91	36.3%	1.05	0.65–1.70	0.834	2.13	0.83–5.47	0.126	1.17	0.79–1.73	0.437
Lymph node status	pN0	147	72.8%	1.09	0.72–1.63	0.690	1.96	0.84–4.57	0.119	1.17	0.84–1.64	0.348
	pN+	55	27.2%	1.10	0.61–1.97	0.747	2.21	0.74–6.64	0.179	1.22	0.75–1.97	0.423
ER status	pos	202	78.0%	1.07	0.74–1.55	0.725	2.03	0.93–4.44	0.072	1.17	0.86–1.58	0.319
	neg	57	22.0%	1.19	0.67–2.11	0.562	1.23	0.33–4.55	0.763	1.16	0.72–1.89	0.547
PR status	pos	142	54.8%	1.16	0.77–1.75	0.478	1.49	0.60–3.70	0.389	1.18	0.83–1.66	0.358
	neg	117	45.2%	1.02	0.65–1.58	0.940	2.29	0.96–5.45	0.063	1.16	0.81–1.66	0.420
HER2 status	pos	53	20.7%	1.02	0.56–1.84	0.957	2.30	0.76–6.92	0.159	1.16	0.71–1.90	0.543
	neg	203	79.3%	1.12	0.78–1.63	0.536	1.64	0.73–3.69	0.233	1.16	0.86–1.58	0.333
Ki67 status	>10%	103	47.7%	0.88	0.55–1.40	0.588	2.12	0.85–5.27	0.115	1.04	0.71–1.51	0.856
	≤10%	113	52.3%	1.25	0.80–1.95	0.325	1.24	0.45–3.44	0.684	1.21	0.83–1.76	0.325
p53 status	pos	57	22.6%	1.27	0.72–2.26	0.412	0.80	0.17–3.73	0.775	1.16	0.71–1.90	0.543
	neg	195	77.4%	1.03	0.71–1.50	0.873	1.84	0.83–4.10	0.133	1.12	0.82–1.52	0.475

Dominant model, TC + CC *vs.* TT; recessive model, CC *vs.* TT + TC; log-additive model, TT = 0, TC = 1, CC = 2; Subgr., subgroup of patients; No., number of patients in each subgroup; OR, odds ratios; 95% CI, 95% confidence intervals; *p*, *p*-values; ER, estrogen receptor; PR, progesterone receptor; HER2, human EGF receptor 2; pre, pre-menopausal; post, post-menopausal; neg, negative; pos, positive; ^a^ patients aged under 55 years or ≥55 years at diagnosis were compared to control subjects of any age.

## 3. Discussion

Here we explored the potential association of two single-nucleotide polymorphisms of the *RECQL2* gene with breast cancer risk. The Arg834Cys SNP is located in the helicase domain of WRN, and the rare Cys variant was shown to exhibit a dramatic reduction of both helicase and exonuclease activities [17]. The Cys (T) allele was previously found in seven of 459 individuals of Spanish ancestry (six heterozygotes and one homozygote), but not in 749 individuals of various other European ancestries nor among 351 African-Americans [17]. In the present study, we genotyped the Arg834Cys SNP in 518 individuals of Austrian (*i.e.*, Western European) ancestry (269 breast cancer patients and 249 controls), and did not find a single Cys allele.

On the other hand, the frequent coding SNP Cys1367Arg in WRN was polymorphic in our study population and could be analyzed for its association with breast cancer risk and onset. We found at borderline significance that the minor C allele was associated with a roughly doubled breast cancer risk in a largely recessive inheritance mode, whereas the breast cancer risk of heterozygotes was hardly increased compared to the common homozygote (TT; Cys/Cys). Moreover, we show for the first time an association of the C allele with an increased fraction of patients developing breast cancer at an age younger than 55. Consistently, pre-menopausal patients had a 3.21-fold increased risk of breast cancer in the recessive inheritance model (95% CI, 1.23–8.36). Since the age at onset this seems to affect the odds ratios associated with Cys1367Arg, we calculated odds ratios adjusted for age in addition to the crude (unadjusted) odds ratios, which increased the magnitude of the observed effects (Table 2). For example, the uncorrected odds ratio for CC compared to TT genotypes was 1.91 (95% CI, 0.89–4.11; *p* = 0.116), whereas the odds ratio adjusted for age was 2.26 (95% CI, 0.98–5.25; *p* = 0.05; Table 2). These findings suggest that the WRN 1367Arg variant might be involved in increased and accelerated breast cancer development in a European population.

RECQL2 is a RECQ DNA helicase that plays a key role in the maintenance of genome stability [9]. Germline mutations in this gene lead to the rare recessive Werner syndrome associated with premature aging and predisposition to cancer [9,11]. The importance of RECQL2 is also supported by recently published work suggesting DNA helicases such as RECQL2 to represent a novel class of targets for anticancer therapy [21]. The *RECQL2* gene is situated in chromosomal region 8p11–12, which contains one or more breast cancer tumor-suppressor genes [22]. RECQL2 has been suggested to serve as a mutator driving tumorigenesis, based on its role in DNA metabolism pathways and the maintenance of chromosomal stability [3].

Epidemiological and functional and analyses have focused on Cys1367Arg, which is located in the COOH-terminal binding domain for TP53 and BRCA1 [23,24,25]. *RECQL2* Cys1367Arg appears to be associated with an increased risk of several age-associated disorders, such as myocardial infarction, diabetes mellitus type-2, and osteoporosis [26,27,28], indicating that this amino acid transition may compromise the functional integrity of RECQL2. Only a few studies have investigated the associations of *RECQL2* polymorphisms with breast cancer. A significant association of the minor allele of Cys1367Arg with familial breast cancer (OR = 1.28; 95% CI = 1.06–1.54) and high-risk familial breast cancer (OR = 1.32; 95% CI = 1.06–1.65) was found in a German study [19]. However, no evidence for an association of Cys1367Arg with the risk of sporadic breast cancer was found in a different study [3]. Moreover, no significant association between Cys1367Arg and increased sporadic breast cancer risk was found in a Chinese study [29]. Together, the discrepancies between these studies may reflect differences in different populations and/or ethnic backgrounds.

To further evaluate a possible association between *RECQL2* Cys1367Arg and breast cancer, we performed a study in Austrian women of exclusively European ancestry. Our findings add a new yet unidentified potential role of this variant in breast cancer. We found that Cys1367Arg was significantly associated with an increased risk of developing breast cancer at an age younger than 55 years, based on recessive (OR 2.76; *p* = 0.021) and log-additive (OR 1.58; *p* = 0.014) genetic models (Table 3). Moreover, comparison of the mean and median age at breast cancer onset and the cumulative breast cancer incidence revealed that patients with the rare CC genotype had an approximately five year younger mean age at onset than TT patients (Figure 1). However, it should be noted that this difference was not statistically significant at the *p* < 0.05 level, which may be due to the small number of CC patients. These data are supported by findings in the subpopulation of pre-menopausal patients, which revealed an OR of 3.21 (*p* = 0.022) under a recessive model. These patients overlap to a large extent with the patients under the age of 55. In addition, we identified a significant association of the 1367Arg variant with pT2–pT4 tumors (tumor diameter >2 cm).

It has been suggested that the tumorigenic contribution of variant WRN in breast cancer could be increased due to accelerated cell growth caused by estrogen [3]. However, no evidence for such a role for Cys1367Arg was found in the same study [3]. We observed only a non-significant trend for an increased breast cancer risk (OR 2.03) associated with the 1367Arg variant in patients with ER positive tumors, which also does not support a role for Cys1367Arg in estrogen-dependent breast cancer development.

In contrast to MYC-driven cancers such as neuroblastoma, where RECQL2 has been reported to provide a critical pro-survival function that is necessary for efficient tumor growth and constitutes a candidate therapeutical target [30,31], our data support the previously described function of RECQL2 as a tumor suppressor of the caretaker type [8]. WS patients are predisposed to cancer [32], suggesting that *RECQL2* could function as a tumor suppressor gene and polymorphisms in *RECQL2* may disrupt this function in breast cancer. In line with this, we observed in our study an approximately twofold risk of breast cancer associated with the CC genotype of rs1346044, which was more pronounced when odds ratios adjusted for age were analyzed (Table 2). Similarly, a German study showed an association between the C allele of rs1346044 and increased familial breast cancer risk [19].

The RECQL2 helicase forms complexes with several other proteins involved in regulating RECQL2 function and many of its interacting proteins bind to RECQL2 via the COOH-terminal domain comprising amino acids 940–1432 [7,9]. The Cys1367Arg polymorphism leads to a non-conservative amino acid exchange. It is near the C-terminus of RECQL2, which binds to both p53 and BRCA1 [33], and near the nuclear localization signal [34]. Although the helicase activity of RECQL2 is only mildly affected, if at all, by the Cys1367Arg SNP [17], it is tempting to speculate that Cys1367Arg may affect breast cancer development by disturbing protein-protein interactions in the COOH-terminal region of RECQL2. Specifically, the 1367Arg variant may affect the binding of p53 and/or BRCA1 to RECQL2, thus subtly compromising their function and leading to a weak phenocopy of BRCA1 and TP53 mutations, namely increased breast cancer risk and a younger age at onset. It has been suggested that 1367Arg might indeed compromise the binding of p53 to RECQL2, thus interfering with the apoptotic function of p53 and increasing breast cancer risk [35]. However, our study revealed no evidence for a significant association of RECQL2 Cys1367Arg genotypes with p53 status, thus our findings provide no support for this hypothesis.

## 4. Experimental Section

### 4.1. Study Population

The study population has been described in detail in [36,37], and its clinical and histopathological characteristics are shown in Table 1. 276 consecutive female breast cancer patients and 255 female controls (patients with benign gynecological lesions and healthy females) of European background were enrolled between 2002 and 2004 at the Department of Obstetrics and Gynecology, Medical University of Vienna (MUV), Austria. This study was approved by the institutional review board of the MUV, and written informed consent was obtained from all participants. rs3087425 (Arg834Cys) genotypes could be determined in 518 subjects (269 cases and 249 controls), which all exhibited the same genotype (Arg/Arg; CC). Thus, this SNP could not be further analyzed. Determination of the rs1346044 (Cys1367Arg) genotype was unsuccessful for four patients and one control, and all analyses of this SNP were based on the remaining 526 subjects (Table 1).

### 4.2. DNA Isolation and Genotyping

Genomic DNA was extracted from blood samples with the QIAamp DNA Blood Midi kit (Qiagen, Venlo, The Netherlands) following the manufacturer’s instructions. Genotyping of SNP rs1346044 (Cys1367Arg; C1367R; c.4099T > C; 108690T > C) located in exon 34 near the C-terminus of RECQL2 protein and rs3087425 (Arg834Cys; R834C; c.2500C > T) located in the central helicase domain was performed by TaqMan PCR with allele-specific, fluorescently labeled probes following the manufacturer’s instructions (Assay-IDs: rs1346044, C_650486_10; rs3087425, C_43158748_10; Applied Biosystems, Brunn am Gebirge, Austria). 40 ng of genomic DNA in a total reaction volume of 10 µL were used.

### 4.3. Statistical Analysis

Statistical analyses were performed with R version 2.15.1 [38]. Chi-square tests with Yates’ continuity correction were used to assess whether the study population deviates from Hardy-Weinberg equilibrium. All 95% confidence intervals plus associated *p*-values were calculated by the mid-*p* exact method. Following a previous recommendation, we did not adjust the results of our subgroup analyses in Table 3 for multiple testing, since these analyses should be considered exploratory [39]. All *p*-values shown are two-sided. *p* < 0.05 was considered significant.

## 5. Conclusions

Epidemiological analyses of the RECQL2 Cys1367Arg are hampered by the rarity of the disease-associated homozygous CC (Arg/Arg) genotype representing only 4.3% of the controls in the present study. Accordingly, many of our findings in the present study attained only borderline significance. However, despite minor limitations in the present study, such as a hospital-based study design, restriction to a limited population, and limited statistical power, our results provide new support for an impact of Cys1367Arg on the putative function of RECQL2 as a breast cancer tumor suppressor. Our results suggest that the rare homozygous CC (Arg/Arg) genotype is associated with an increased risk and early onset of breast cancer. In contrast, this substitution was not significantly associated with breast cancer risk in subpopulations other than premenopausal patients and patients younger than 55 years at onset. Further epidemiological studies and/or meta-analyses with larger, ethnically-diverse populations as well as functional evaluation of the RECQL2 Cys1367Arg polymorphism are warranted to further address these questions.
